# Effect of different weekly frequencies of Chen-style Tai Chi in elders with chronic non-specific low back pain: study protocol for a randomised controlled trial

**DOI:** 10.1186/s13063-022-06909-2

**Published:** 2022-11-22

**Authors:** Ruihan Wan, Jian Shi, Kun Hu, Yafei Wang, Xue Jiang, Wangwang Yan, Mali Cao, Yuling Wang

**Affiliations:** 1 College of Rehabilitation Medicine, Fujian University of Traditional Chinese Medicine, Fuzhou, China; 2 Rehabilitation Medicine Center, The Sixth Affiliated Hospital of Sun Yat-sen University, Guangzhou, 510655 China; 3 College of Kinesiology, Shenyang Sport University, Shenyang, China; 4 Department of Sport Rehabilitation, Shanghai University of Sport, Shanghai, China; 5 Department of Rehabilitation, Changsha Social Work College, Changsha, China

**Keywords:** Tai Chi, Chronic Low back pain, Old people, Different frequency, Randomised controlled trial

## Abstract

**Background:**

Tai Chi (TC), as one of mild to moderate exercise therapies specifically recommended by clinical practice guideline from the American College of Physician, is a viable option for chronic non-specific low back pain (CNLBP) treatment. Nevertheless, limited studies focused on the effect of different weekly frequencies of TC in elders with CNLBP. This superiority study aims to compare the differences of TC with different weekly frequencies in elders with CNLBP on the premise of proving its effectiveness, and identifying whether mindfulness mediates the effect of TC on treatment outcomes.

**Methods:**

In total, 284 senior citizens with CNLBP will be recruited in this single-centre, randomised, single-blinded (outcome assessors, data managers and the statistician), parallel controlled trial. Participants will be randomly divided into either one of three TC groups (1, 3, or 5 sessions/week, on the basis of weekly health educational lectures) or weekly health educational lectures, sustaining for 12 weeks, followed by 12 weeks of follow-up after the end of intervention. The primary outcome (the changes of LBP intensity at rest) will be measured at baseline before randomisation and immediately after the completion of weeks 4, 8 and 12 of the intervention, and the end of follow-up (week 24) using the visual analogue scale (VAS, 0–10 cm) to put a mark on the VAS scale to show how severities of their average low back pain have been over the past 24 h. Secondary outcomes, including Beck Depression Inventory-II, Pain Catastrophising Scale and Five Facet Mindfulness Questionnaire, Oswestry Disability Index and Short Form-36, will be measured at baseline and immediately after the completion of week 12 of the intervention and end of follow-up. The intention-to-treat and per-protocol principles will be used to analyse outcomes with a setting at *α* = 0.05 as statistical significance.

**Discussion:**

This comprehensive and detailed protocol will be the first trial to compare the effectiveness of different weekly frequencies of TC in elders with CNLBP. The outcomes may provide valuable data about the choice of the ideal number of sessions to further normalise the application of exercise for clinicians.

**Trial registration:**

Chinese clinical trial registry ChiCTR2200058190. Registered on 1 April 2022.

**Supplementary Information:**

The online version contains supplementary material available at 10.1186/s13063-022-06909-2.

## Introduction

Pain occurring below the costal margin and above the buttock folds can be defined as low back pain (LBP). The majority of patients with LBP have a group of clinical subtypes collectively known as non-specific low back pain (NLBP), in which most of the events have no definite causes [1] and chronic NLBP (CNLBP) is NLBP that lasts 12 weeks or longer [2, 3]. The lifetime prevalence rate of LBP is 84%, amongst which CNLBP accounts for almost one third of cases, especially in elders [4], affecting nearly 20–25% of the population older than 65 years. Although LBP is not a fatal disease, it is the foremost leading cause of physical disability worldwide [5], ranking sixth in the overall cause of disease burden (disability-adjusted life years) [2]. Pain induces serious psychological or medical pathology, which incurs a huge burden on society in terms of medical health and lost productivity. Furthermore, identifying effective non-pharmacological approaches for old people with CNLBP is urgently needed because of the risks of surgery and drug abuse. However, specific novel alternative treatments for pain are still unclear. Hence, although the effect size of traditional interventions is moderate [6, 7], these are still worthy of further exploration.

Tai Chi (TC) is one of mind-body exercise therapies specifically recommended by clinical practice guideline from the American College of Physician [6] and is broadly applied to different age groups worldwide [8]. The latest American Physical Therapy Association guidelines on LBP management in elderly also recommended a variety of general exercise training for conservative treatment. An exercise training should restore or improve the overall strength or endurance of major muscle groups in the upper/lower extremities and trunk; such exercises include exercises for flexibility/mobility and aerobic/conditioning exercises [9], which indicates TC may be a CNLBP-modifying treatment option. Meanwhile, pain relief has been found to play an important role in the movement and quality of life (QOL) of patients suffering from LBP [10]. TC combines slow, gentle movements with deep breathing and mental focus, can fully activate abdominal core muscles, improve pulmonary ventilation in patients with LBP [11], strengthen the stability of lumbar bone structure [12] and promote the improvement of lumbar proprioception [13, 14]. To a certain extent, it may alleviate pain, pleasure, improve participants’ self-efficacy and mitigate CNLBP patients with anxiety and/or depression mood.

A majority of previous studies chose TC intervention in patients with chronic pain for 60 min at a time for 12 weeks is relatively common [15–18]. Patients with LBP who have comorbidities are eager to relieve symptoms as quickly as possible. For elders without TC foundation, completing a set of integrated TC learning in a short time is unrealistic [16, 19]. Hence, selecting a part of core muscle with high activation levels for specific familiarisation and training in the preliminary exercise can shorten the learning time of TC and may be more in line with the psychological expectations of patients for symptom improvement efficiency. Chen-style TC with 16 forms may be an optimal choice for the elderly. It has the following characteristics. Firstly, it can be readily understood by elders. Secondly, the difficulty of movement gradually increases, and weight bearing gradually changes from bilateral support to unilateral support. Thirdly, the speed of movements constantly changes, which may increase the interest in TC training. However, whether different weekly frequencies of TC training (i.e. fewer or more sessions in the same week) may relieve pain in the elderly is still unknown, and pain trajectories have not been identified.

Some studies showed TC to be effective for treatment of CNLBP, reporting positive outcomes such as reduction in pain or psychological distress such as depression and anxiety, reduction in pain-related disability and improved functional ability [16, 17, 20, 21], but few studies have focused specifically on older adults especially older people with CNLBP. Given the promising data from younger adults [20, 21], the value of TC for older adults with CNLBP remains worthy of investigation. Only two studies have looked at the role of TC in the elderly with CNLBP [22, 23], but both have some limitations. Lee et al.’s study [22] demonstrated the multifaceted benefits of TC for the elderly with CNLBP, but this study is only a qualitative study and the sample size is seriously insufficient (*n*=18). Sherman et al.’s study [23] confirmed that TC intervention is feasible and acceptable in the elderly with CNLBP, but in terms of sample size, it has the same methodological problems as the above studies. Besides, its primary outcome did not focus on the effect of TC on pain relief in elderly patients with CNLBP. A meta-analysis showed that there were significant heterogeneity and quality differences in the results of TC intervention with LBP, which may be caused by differences in dose (such as frequency, duration, course of treatment and mode) [18], which is also potentially suggestive that the dose of TC is an important factor affecting its effectiveness. Recent meta-analyses related to TC have also revealed that researchers should concentrate more on the timing and frequency of TC training, in order to make clearer claims about its beneficial effects [24, 25]. The lack of information has led to the severe under-utilisation of conventional exercise by clinicians and the poor exercise compliance of patients.

In light of the abovementioned studies, the primary objective of this study will compare the differences in the curative effects of TC with different weekly frequencies in elders over 60 years [26, 27] with CNLBP on the premise of proving its effectiveness. Besides, we will also identify whether mindfulness mediates the intervention effect of TC. We hypothesised that high weekly frequencies (five times a week) of TC intervention may be more beneficial to pain, physical and psychosocial functioning and QOL in elderly with CNLBP than low (once a week) and moderate weekly frequencies (three times a week) TC intervention, and that the higher the baseline mindfulness level of participants, the better the effect of TC on CNLBP symptoms. The interventions are relatively safe. The results of this trial are expected to help clinicians and future researchers provide references in selecting the ideal TC dose and standardise the formulation of TC exercise prescription for patients with CNLBP, and enrich the theoretical basis for TC dose selection. For patients, we expect the results of this study to provide evidence-based advice for treatment planning for patients with CNLBP and to use TC as an ideal form of self-care to relieve their symptoms and improve QOL.

## Methods and analysis

### Trial design

This study is a single-centre, randomised, single-blinded (outcome assessors, data managers and the statistician), parallel controlled trial, which will be carried out in Changsha First Social Welfare Institute, Hunan Province, China. In total, 284 eligible participants with CNLBP will be randomly and equally divided into four groups in a 1:1:1:1 allocation ratio. Three experimental groups will undergo 12 weeks of supervised TC training and complete one, three or five TC sessions weekly, respectively. The control group will not receive any rehabilitation programme. Meanwhile, all participants will receive weekly health lectures until the end of the 12-week intervention and will be required to maintain a healthy lifestyle, afterwards followed by 12 weeks of follow-up after the end of the intervention.

**Fig. 1:**
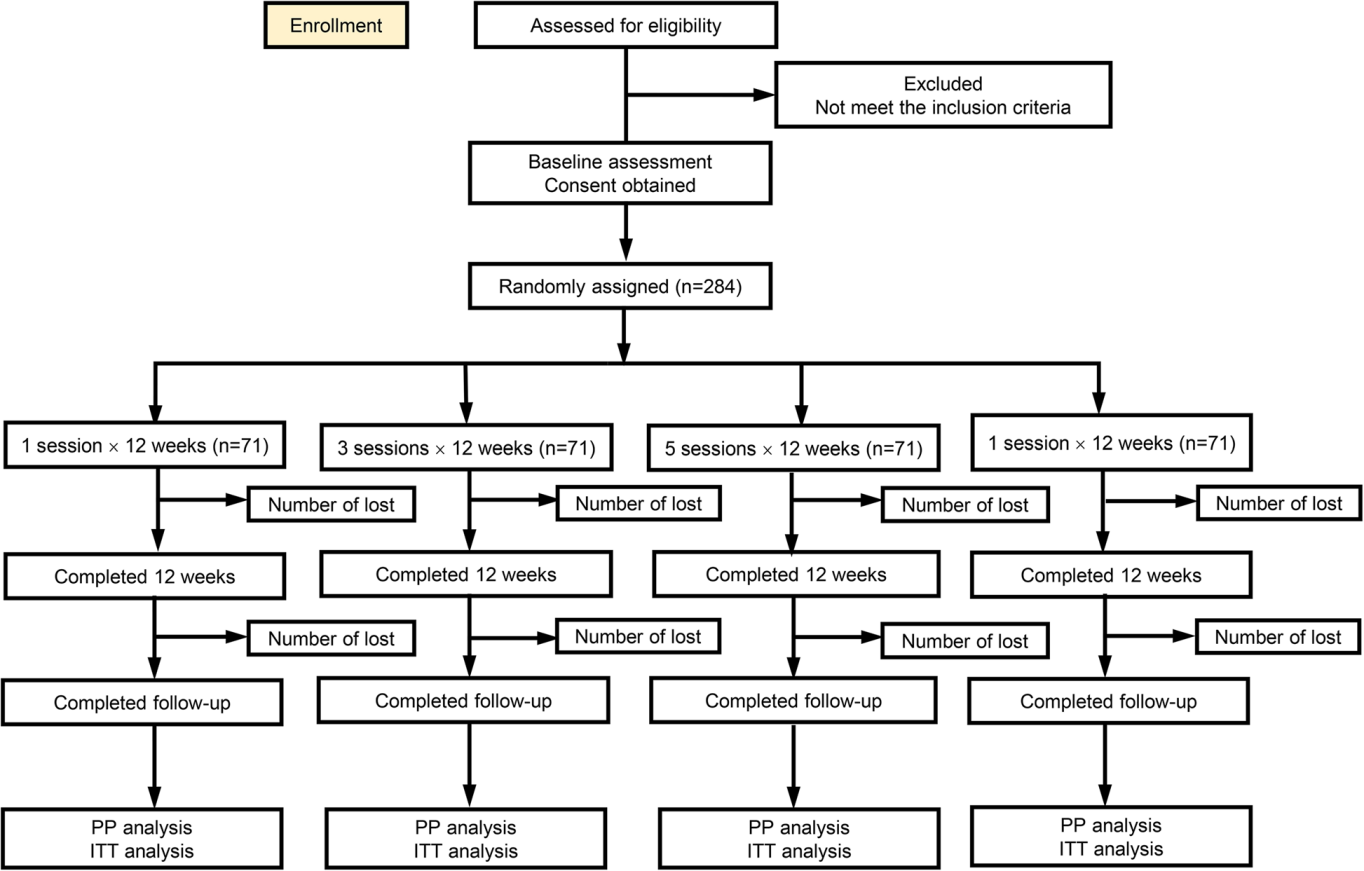
Flowchart of this trial. Notes: PP: Per-protocol; ITT: Intention-to-treat

The primary outcome (the changes of LBP intensity at rest) will be measured at baseline before randomisation and immediately after the completion of week 4, week 8, week 12 of the intervention and the end of follow-up (week 24). The secondary outcomes will be measured at baseline before randomisation and immediately after the completion of week 12 of the intervention and the end of follow-up (week 24). The details of the time schedule of this trial are shown in Table 1. The recommendations of the Consolidated Standards of Reporting Trials (CONSORT) will be strictly followed, and the protocol will be reported in line with the Standard Protocol Items (SPIRIT) [28] (Additional file 2). The flow chart is presented in Fig. 1.

**Table 1:**
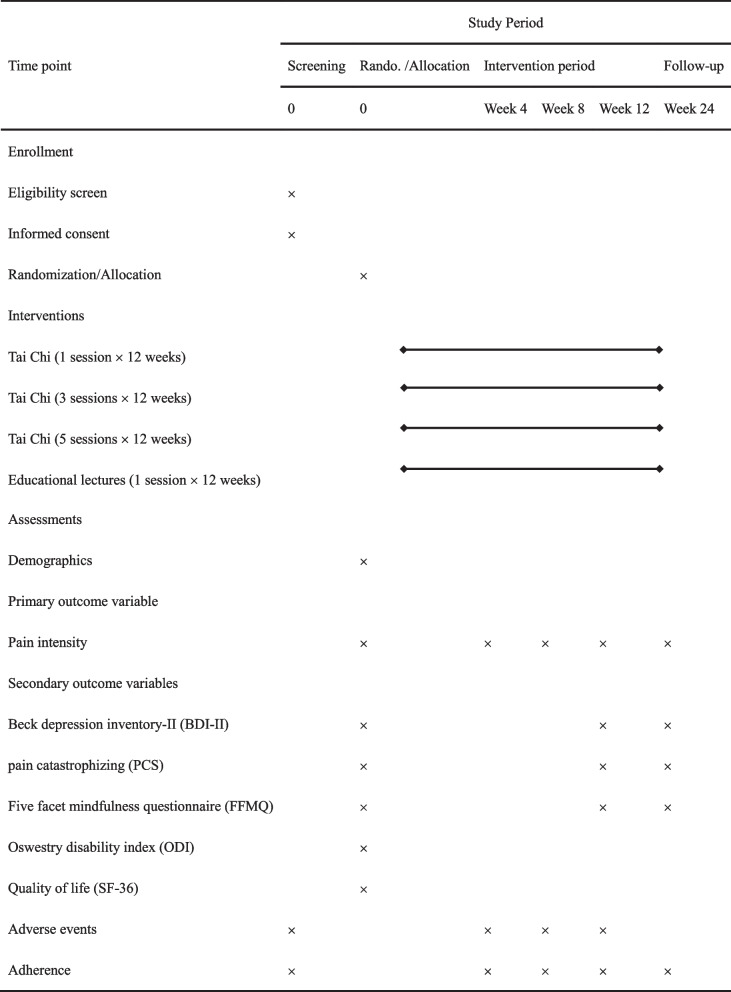
The schedule of enrollment, interventions and assessments according to SPIRIT items

### Patient and public involvement

The involvement of the public and patients is becoming a necessary part of health research [29]. Patients are often more aware of their disease and lifestyle needs than many medical professionals. They also have important ideas about which studies will be most beneficial to their lives, especially about how symptoms can be managed in a way that improves the QOL. We are inviting patients who have experienced or are experiencing CNLBP and those who are interested in the study, to join our community of interest (through social media, and by putting up recruitment posters outside Changsha First Social Welfare Institute, Hunan Province, China). The public and patient representatives (PPR) will be involved during the planning, conduction and analysis of the trial. The PPR group will meet every 2 months to provide peer support and share personal experiences.

### Participants

#### Recruitment and ethics

The enrollment programme will run from the fourth quarter of 2022, and follow-up work is expected to be completed in October 2023. A total of 284 participants will be recruited via the hospital bulletin and official website of Changsha First Social Welfare Institute and a wide range of media channels, including local advertisements and WeChat (a free popular communication application in China).

Any interviewee who volunteers to participate in the study will be requested to fill a screening assessment to identify their eligibility. The participants will be informed of the study details, such as interventions, potential benefits, specific training schedule (including date and time) and some announcements to avoid potential risks and control confounding factors. In addition, all participants will sign informed consent before the study begins.

This study was approved by the Ethics Committee of Changsha Social Work College (CSMZ2022002) and registered at the Chinese Clinical Trial Registry (ChiCTR2200058190). It will be carried out in accordance with the Declaration of Helsinki.

#### Eligibility criteria

This trial will include the following: participants who are right-handed; adults aged 60 years or above [26, 27]; diagnosed with CNLBP for at least 3 months in line with the CNLBP definition of the National Institutes of Health (NIH) [30]; VAS score ≥ 4 [31]; Mini-Mental Status Examination (MMSE) score ≥ 24 [32]; no habitual regular exercise and TC training history in the past 6 months; able to ambulate independently and participate in TC training.

The exclusion criteria include the following: LBP caused by mandatory spondylitis, malignant tumours, vertebral fractures and spinal infection; serious diseases that limit ability to participate in TC training, including dementia, neurological disease, symptomatic heart or vascular disease (angina, peripheral vascular disease, congestive heart failure and severe hypertension), recent stroke, severe insulin-dependent diabetes mellitus, psychiatric disease, renal disease, liver disease, active cancer or anaemia; having analgesic drugs or regular physical therapy programmes for CNLBP within the past year; participating in a similar study within the past year and could have significant impact on the results of this study; planning to move permanently from the area during the trial period.

The withdrawal criteria comprise occurrence of severe adverse events (e.g. nausea, vomiting, palpitations) during the study and patients deemed unsuitable to continue the trial by specialist doctors; and voluntary withdrawal from the trial for any reason at any time.

#### Sample size

The sample size was estimated using the PASS software (ver. 15.0). Limited studies have focused on the effect of different weekly frequencies of TC in elders with CNLBP. As such, the primary indicator was the changes of LBP intensity at rest (represented as VAS, score of 0–10). The sample size was calculated via the minimum clinically important difference of 2 on the VAS of pain intensity, and expecting the standard deviation of 2.2 immediately following TC training [33–35]. Considering the multiple comparison, the Bonferroni adjustment was applied, a sample with 240 participants was needed with a power of 80% and an alpha of 0.05 (*α*=0.05 is the overall allowable type I error in the comparisons between each experimental group and the control group sequentially). Moreover, considering a 15% attrition rate, at least 71 participants per group will be needed in this protocol trial [Total participants: 240/ (1−0.15) ≈ 284 participants, considering an equal allocation of 1:1:1:1 to four groups].

### Randomisation and blinding

The simple stochastic method will be applied in this study. SPSS 24.0 (IBM, Inc., Chicago, USA) software will be used by a statistician who is not involved in this study to generate a random number sequence. Random numbers with the group allocation code will be kept in opaque and sealed envelopes, which will be prepared by a person who is not involved in the whole process of the study, including recruitment, enrollment, evaluation and statistical analysis of data and random assignment the participants to the four groups (word “1” or “2” or “3” or “4”) with equal probability according to the order of entry. Random assignment results in all enrolled eligible participants will be informed by telephone after completing baseline assessment data collection and signing an informed consent form.

Considering the nature of TC, blinding the participants and TC coaches during the process of TC training is completely impossible. Hence, outcome assessors, data managers and the statistician will be blinded in this trial, and the blind will be kept by the custodian of the random sequence, who is not participating in the process of participant recruitment, intervention, outcome evaluation and data statistical analysis. Upon completion of all data analysis, the random sequence custodian will reveal the treatment allocations.

### Qualification of practitioners

Only licensed TC coaches with more than 10 years of teaching experience will perform TC training. Each coach will receive additional human subject protection training a week before the TC training begins. Furthermore, each intervention group will be assigned a registered nurse with at least 3 years of clinical experience to avoid serious adverse events. Every participant will be guided in standardised operating procedures before study initiation to further clarify the protocol trial, flow chart and individual responsibilities.

### Study intervention

#### Experimental group: TC training

The trial protocol will be administered at the Sports Centre of Changsha First Social Institute. A total of 284 eligible participants will be randomly divided into four groups. Amongst these, three groups (group 1, group 2 and group 3) will undergo TC training with different frequencies (one, three or five sessions/week, 60 min/session, sustain for 12 weeks). Before the training begins, each group will be assigned a coach. The participants will be familiarised with the TC programme, including principles, practice strategies and safety considerations, through printed materials and short videos a week prior to study initiation. Three research assistants will supervise and guide the participants’ learning conditions through weekly phone calls.

**Fig. 2:**
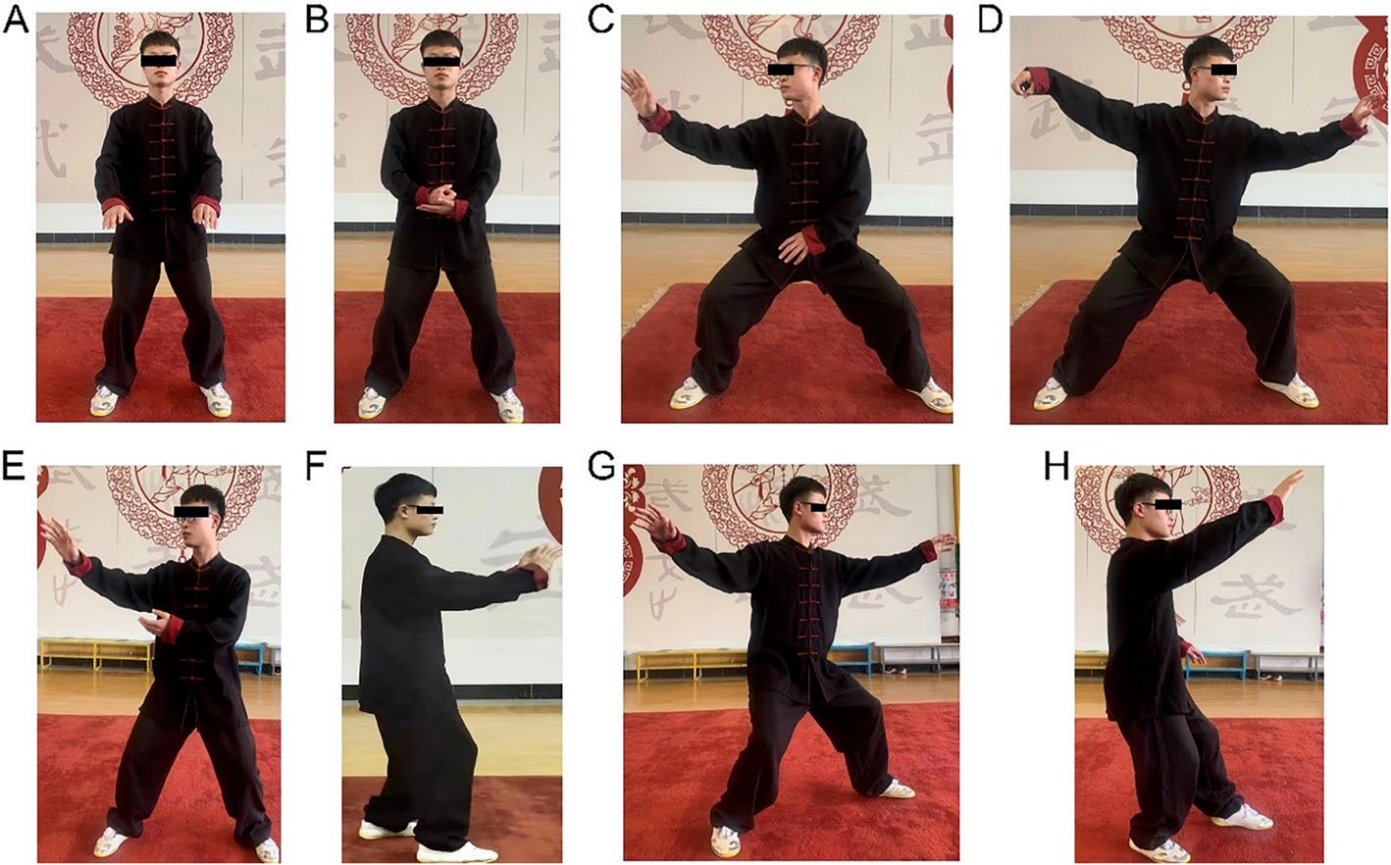
Sixteen-form Chen-style Tai Chi. Notes: **A** Commencing Form. **B** Buddha’s Warrior Attendant Pounds Mortar. **C** Tuck in Robes. **D** Single Whip. **E** Wave Hands Like Clouds. **F** Double Push Palms. **G** Step Back and Whirl Arms on Both Sides. **H** White Crane Spreads Wings

**Fig. 3:**
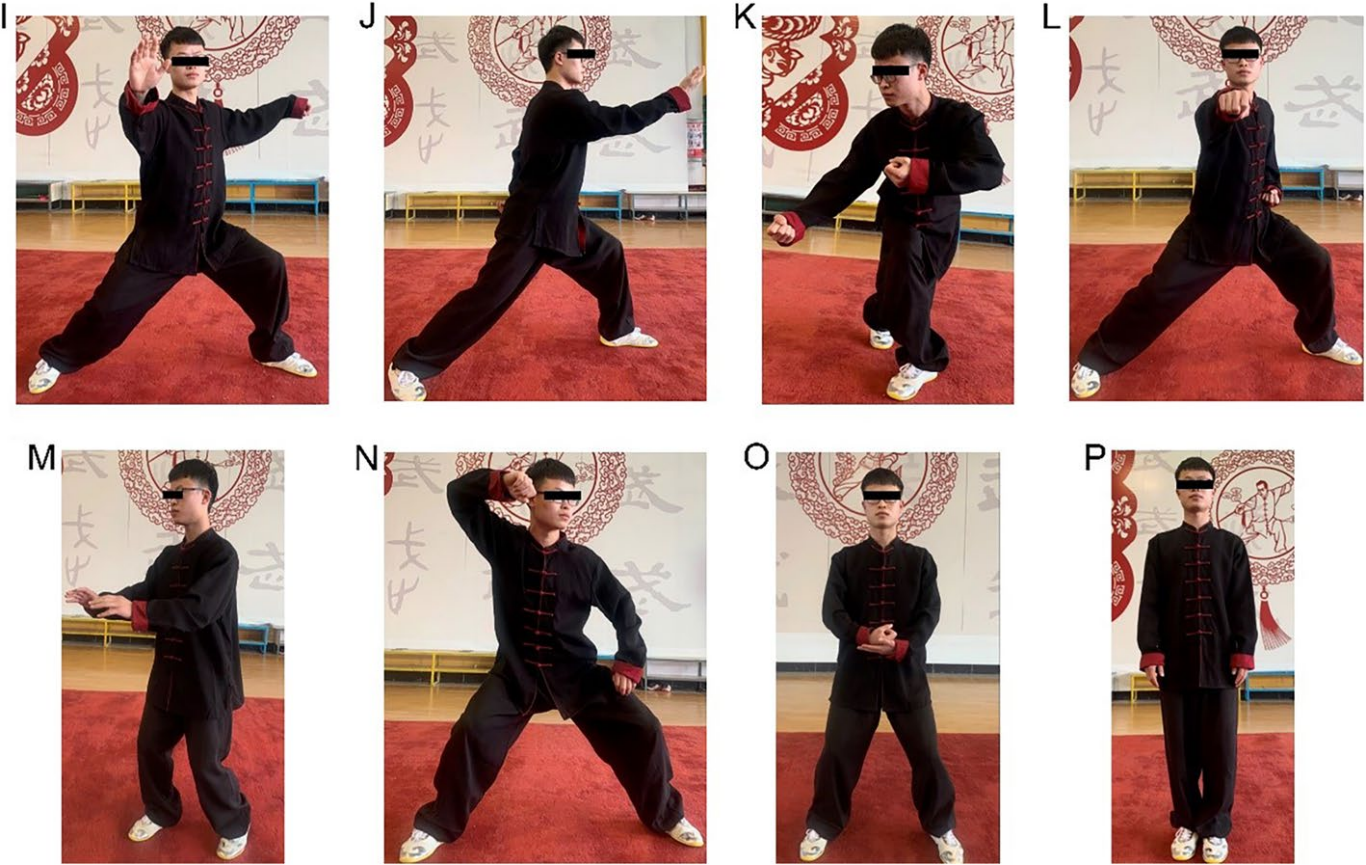
Sixteen-form Chen-style Tai Chi. Notes: **I** Diagonal line spread step. **J** Deflect through The Back. **K** the Chopping Hand. **L** Strike Fist. **M** Six Seals and Four Closings. **N** Single Whip and Body Defending Punches. **O** Turn-back and Buddha’s Warrior Attendant Pounds Mortar. **P** Closing Form (mode: Mr. Haiyan Kou)

At the beginning of this study, participants of three TC groups will commence the interventions at the same time under the guidance and supervision of coaches to prevent seasonal changes in disease severity. Group 1 will practice Chen-style TC one time a week for 12 weeks under the guidance of a coach with 10 years of teaching experience. The TC training programme shall be jointly formulated by the research group members and coaches after discussion. See Figs. 2 and 3 for the movements included in TC. The whole training process includes 10 min of warm-up, 40 min of TC training and 10 min of cooling down. The details are shown in Additional file 1. Sessions for the first week are designed to enable participants to master each training movement, breathing technique and relaxation method under the guidance of the instructors. The remaining sessions will consist of the following sections: (1) warming-up and review of TC principles and techniques; (2) Chen-style TC with 16 forms for two times; (3) breathing mechanics and (4) cooling down. Meanwhile, video recordings will be used to monitor the quality of TC training and offer feedback to coaches on time throughout the study. For group 2 and group 3, the parameters will be in line with group 1, except that training frequency will be transferred to 3 times a week and 5 times a week, respectively.

#### Control group: health education

The control group will keep their daily lifestyle and will not receive any rehabilitation treatment during the intervention, except for one health lecture per week for 12 weeks.

#### Permitted and prohibited concomitant treatments

Participants should avoid performing any regular physical exercise based on the Centres for Disease Control and Prevention guideline (https://www.cdc.gov/physicalactivity/basics/older_adults/index.htm), except TC training, throughout the course of this protocol trial. Besides, participants still can maintain their regular drug treatments for some diseases (e.g. diabetes, heart disease and hypertension), but painkillers, such as opioids, non-steroidal anti-inflammatory drugs and antidepressants, will be banned. Any changes in drug usage throughout the intervention period will be recorded.

### Outcomes

Primary and secondary outcomes will be evaluated at baseline, immediately after finishing the TC training (week 12) and at the end of the follow-up period (week 24). In addition, primary outcome (the changes of LBP intensity at rest) will also be collected every week 4 and week 8 after the intervention started. All indicators will be selected in assessing outcomes for estimating treatment success for CLBP [36–38], concentrating on pain intensity, psychology, QOL and functioning disability.

#### Baseline characteristics of trial participants

The socio-demographic information that will be collected includes gender, age, body mass index, marital status, living status, working status and education level. The clinical and CNLBP-related information that will be collected includes LBP duration, self-reported comorbidities, drug use condition (type, frequency and duration) before the start of this trial and history of back surgery.

#### Primary outcome

The primary outcome (the changes of LBP intensity at rest) will be measured using the VAS [39, 40], a horizontal line of 0–10 cm long, scores of 0 and 10 are at the extremes of the two scales, representing the pain status: “no pain” and “worst imaginable pain,” respectively. Participants will be asked to put a mark on the VAS scale to show how severities of their LBP have been over the past 24 h [39]. The higher the VAS score, the greater the pain intensity. Such has confirmed its sensitivity and reliability for patients with LBP [41]. The outcome will be collected at baseline before randomisation and immediately after the completion of week 4, week 8, week 12 of the intervention and the end of follow-up.

#### Secondary outcomes

The comorbidities of CNLBP will be considered to avoid the effect of confounding factors on outcome indicators. For secondary outcomes, we will collect a wide range of variables, including psychology (Beck Depression Inventory-II [BDI-II], Pain Catastrophising Scale [PCS] and Five Facet Mindfulness Questionnaire [FFMQ]), functional disability (Oswestry Disability Index [ODI]) and QOL (Short Form-36 [SF-36]) as follows:

- BDI-II: BDI-II contains 21 items and is widely used to measure the severity of depression symptoms. A higher BDI-II score indicates a greater degree of depression. Its Chinese version has been proven to have good reliability and validity [42].
- PCS: PCS is used to describe pain catastrophising. It consists of three subscales with 13 items: rumination, magnification and helplessness. Each item is scored from 0 (never) to 4 (always). A higher score represents a higher level of pain catastrophising [43]. The psychometric properties of the Chinese version of PCS are satisfactory and have good reliability and validity [44].
- FFMQ: This scale is applied to measure five aspects of mindfulness: observe, describe, act aware, nonjudge and nonerect. The questionnaire contains 39 items; each item is scored on a scale of 1 to 5; a higher score indicates a higher level of mindfulness [45]. The Chinese version of the FFMQ has acceptable psychometric properties and is a valid instrument for the assessment of mindfulness [46].
- SF-36: This scale evaluates health-related QOL from eight aspects with 36 items (0–100), including physical functioning, role–physical, bodily pain, general health, vitality, social functioning, role–emotional, mental health and health transition. A higher score indicates a greater health status [47].
- ODI: This scale assesses disability in patients with LBP on 10 dimensions, including pain intensity, personal care, lifting, walking, sitting, standing, sleeping, sexual activity (if applicable), social life and travelling. Each dimension has six levels. A score of 0 was used for the minimum disability level; five points were used for the highest degree of disability. The Chinese version of the ODI is responsive and appropriate for usage in patients with CLBP after conservative therapy [48].

### Adherence

The electronic data management platform Yiducloud will be set by us to regularly automatically release daily tasks to participants via WeChat. A research assistant will be responsible to ensure that each participant is informed. The contents of the notice include the following aspects:

- Training plan for the next day;
- Reminders to avoid performing a new regular exercise programme except the TC training and maintain their usual physical activities as much as possible;
- Monitoring sign-in and recording medicine changes timely.

In addition, the research assistant will also be responsible for monitoring the participants’ attendance and filling out the class sign-in sheets for each in-person lesson, which will be used to track the participants’ attendance.

### Safety evaluation

Regardless of the reason for withdrawal during the process of intervention and follow-up period, we will figure out and detailed record the reasons for the withdrawal of participants in the case report forms (CRFs). If the participants experience any adverse events (AEs, defined as any impairment caused by TC training), the nurses will handle the AEs at once and contact the relevant research assistant. The details of AEs, including the time of occurrence, duration, degree of symptoms and treatment measures, will be recorded by the assistant in CRFs.

The doctor will provide comprehensive consideration to evaluate their correlation with clinical intervention training, who will advise whether the participant could continue to participate in the following study and give any medical treatment to relieve uncomfortable symptoms. Meanwhile, such events will be reported by the Ethics Committee. Then, the assistant in CRFs will calculate and record the incidence of adverse events via the following formula: Incidence of AEs (%) = (sessions with AEs / total exercise sessions in this group) × 100%, to measure the safety concerns. In addition, we will try to contact such participants and expect them to complete the follow-up.

### Data management

Clinical data will be collected through printed CRFs, which will be stored in locked filing cabinets. Two independent research assistants who are not involved in the trial will enter the data into the electronic data management platform Yiducloud (https://www.yiducloud.com.cn/) for data imputation and check the data to monitor accuracy. All paper files and electronic documents can only be consulted by authorised researchers.

### Oversight and monitoring

The Human Research Ethics Committees review all study activities semiannually, which consists of three professors and five assistant professors who have no conflicts of interest in the study. Meanwhile, they can initiate an independent study audit at any time to ensure the quality of this study, mainly including recruitment; the accuracy, completeness, and clarity of data; attendance; adverse effects, and exercise frequency. These contents will be recorded in the shape of forms, videos, and pictures and sent to the Project Management Group (consisting of principal investigator [PI], study coordinators, coaches, a doctor or nurse with relevant disease expertise) in time. The Project Management Group will be required to give realistic feedback within four weeks. Amongst these, the PI is responsible for supervising the trial. The study coordinator is in charge of monitoring the study’s progress, such as recruitment, intervention, data fidelity, adverse effect, and providing day-to-day support for the trial. Coaches are responsible for establishing the criteria of TC. The doctor and nurse are in charge of providing safety advice and first treatment in case of adverse effects. The Trial Steering Committee consists of the statisticians, PI, the sponsor, the public, and patient representatives, which supervises the overall conduct of the trial. In addition, any information about this trial will be discussed biweekly by the Project Management Group during regular supervision meetings; if the study involves significant modifications and decisions, the Trial Steering Committee must be informed and attended. An independent Data and Safety Monitoring Board (DSMB) will be set up to review the trial data every 4 weeks to monitor data progress to ensure accuracy and safety, which consists of specialists with expertise in chronic pain clinical trials, statistical design and mind-body exercises who have no conflicts of interest in the study. Besides, we do not anticipate any major protocol amendments to this study. If this study has one, communication of significant protocol amendments within the scope of the study will be the responsibility of the PI. Information will be distributed by the project regulatory specialist or research assistant on behalf of the PI.

### Statistical analysis

The SPSS 24.0 software will be used to perform statistical analysis, and the primary outcome is the changes of LBP intensity at rest (week 12 minus baseline [week 0]). Firstly, we will compare the differences between each experimental group and the control group sequentially via using analysis of covariance (ANCOVA), adjusted for the baseline values of the VAS pain scores. The significance level of primary outcome will be set as *α* = 0.0167. If significant differences are observed amongst the three experimental groups, we will use the fixed-sequence test to compare the differences between TC group (1 session/ week) and group (5 sessions/ week); group (1 session/ week) and group (3 sessions/ week); group (3 sessions/ week) and group (5 sessions/ week) sequentially using analysis of covariance, and the significance level of fixed order test will be set as *α* = 0.05. Treatment effects with corresponding 95% confidence intervals will be presented. The intention-to-treat (ITT) will be used to analyse the primary and secondary outcomes when participants have completed randomisation and received treatment for at least 4 weeks. Then, the per-protocol (PP) analysis of the primary outcome will be used as a sensitivity analysis when participants have completed 12-week intervention and have a compliance rate of > 85%. The results of ITT and PP analysis will be compared to check the consistency of results. The missing data will be adjusted by the multiple imputation method. In addition, multivariate regression analyses will be performed to examine the effect of the TC training on the primary outcome measure, adjusting for variables in the baseline data, such as age, sex, and the level of physical activity, to verify the consistency of the results between analysis of covariance and multivariate regression analysis, and further to improve the reliability of the results. Mediation analysis will be performed through a series of linear regression models to evaluate the strength of the relationships amongst the treatment/control group, the mediator and pain outcome to identify whether baseline mindfulness of participants mediates the effect of TC on treatment outcomes.

## Discussion

To our knowledge, this study protocol is the first protocol for a RCT that will investigate the effect of different weekly frequencies of Chen-style TC training in elderly with CNLBP. Developing effective and well-received non-pharmacological approaches to improve pain, disability and QOL in elderly with chronic pain is a national priority [20, 49]. The study will explore whether TC training could be considered a complementary and alternative medical therapy for pain reduction in elders with CNLBP and further investigate minimal effective frequency by comparing different weekly frequencies of TC training. In addition, this study will explore whether baseline mindfulness could be associated with pain reduction from TC training in CNLBP.

As a popular traditional exercise in China, TC has been widely used in chronic pain, such as fibromyalgia [50], knee osteoarthritis [51] and CNLBP [52]. However, the underlying mechanisms on TC training for improving pain and physical function are still unclear and may be ascribed to altered central elements. Long-term TC practice can induce regional structural changes in the precentral gyrus, insular sulcus, and middle frontal sulcus [53]. A previous RCT also found moderate to high correlations between TC-associated pre-post changes in the amygdala–medial prefrontal cortex functional connectivity, as well as pain and physical function improvement [54]. These studies suggest that TC may directly affect the cerebral cortex to regulate pain and physical function through regular practice.

Furthermore, TC pays attention to physical movement and emphasises the state of syncretism of the body and mind [55]. A more positive psychological state corresponds to a higher effective participant adherence [56, 57]. Previous data showed that the co-occurrence of CNLBP and emotional and cognitive factors (e.g. depression or anxiety disorders) is prevalent with a coexistence rate of up to 30–60% [58]. Pain induces serious psychological or medical pathology and incurs a huge burden on society in terms of medical health and lost productivity [59]. Evidence from meta-analysis revealed that cognitive functioning as a positive association with chronic pain and is one of the leading reasons for the generation, persistence and development of chronic pain [60]. Hence, our study will collect psychological outcomes, including BDI-II, PCS, FFMQ, ODI, and SF-36, to avoid the influence of confounding factors.

Notably, mindfulness, as a complex psychosocial variable and a predictor for better health outcomes from exercise, should be paid more attention. Poor adherence and low pain-coping skills are regarded as the major hindrances to the effect of exercise in CNLBP, and mindfulness has been highly associated with pain-coping skills [61, 62] and superior adherence [63, 64]. A recent study indicated that regardless of the effectiveness of exercise, adherence in patients with CLNBP always declines considerably over time [65], which is probably closely related to improving the effectiveness of TC interventions in chronic pain. In addition, Hall et al.’s study [66] provided initial evidence that pain catastrophising, as one of the cognitive appraisal outcomes, has partial mediation, that is, it reduces about 1/3 of pain intensity and 2/3 of disability. Notably, a strong link between baseline mindfulness and the level of pain catastrophising has been verified in a previous study [61]. Lee et al.’s study also confirmed this relationship in patients with knee osteoarthritis, which seems to suggest that higher mindfulness corresponds to a better response to TC training [67]. Up to now, limited study has investigated the longitudinal regression and correlation of mindfulness on the therapeutic effect of TC. Hence, our study makes reasonable speculation that baseline mindfulness may also influence the effect of TC in elderly with CNLBP. The results will contribute to further understanding the heterogeneity of TC response in patients with chronic pain and help physicians to develop personalised exercise prescriptions for such patients.

However, this trial still has several potential limitations and challenges. Firstly, although we will conduct blinding as much as we can, no blinding is possible for coaches and participants in terms of treatment allocation, which is also an inherent limitation of such studies [17, 68, 69]. Secondly, although we will hire experienced coaches and establish a strict quality control programme for participants (e.g. monitor practice and sign-in), the degree of treatment efficiency may become uneven depending on the teaching level of each coach and the acceptability of participants. In short, we will standardise and normalise every step of the study as much as possible to ensure that high-quality evidence is obtained.

Overall, this protocol will be the first RCT of TC intervention designed for the elderly with CNLBP. It will fill the gaps of the evidence on the different weekly frequencies of Chen-style TC in elderly with CNLBP. Furthermore, the trial can provide some mirror for judging whether TC training is appropriate for clinical application through a comprehensive assessment of the dose of TC training.

### Trial status

The trial will begin to recruit in November 2022 and follow-up work is expected to be completed in October 2023.

## Supplementary Information

**Additional file 1.** Instructions for Tai Chi.

**Additional file 2.** SPIRIT checklist.

**Additional file 3.** Informed consent document.

**Additional file 4.** Sample size calculation.

## Data Availability

The datasets analysed during the current study and statistical code are available from the corresponding author on reasonable request, as is the full protocol.

## Abbreviations

TC: Tai Chi
LBP: Low back pain
NLBP: Non-specific low back pain
CLBP: Chronic low back pain
CNLBP: Chronic non-specific low back pain
QOL: Quality of life
CONSORT: Consolidated Standards of Reporting Trials
SPIRIT: Standard Protocol Items
PPR: Public and patient representatives
NIH: National Institutes of Health
MMSE: Mini-mental status examination
BMI: Body mass index
VAS: Visual analogue scale
PCS: Pain catastrophising
ODI: Oswestry Disability Index
FFMQ: Five facet mindfulness questionnaire
SF-36: Short Form-36
BDI-II: Beck depression inventory-II
AEs: Adverse events
CRFs: Case report forms
PI: Principal investigator
DSMB: Data and safety monitoring board
ITT: Intention-to-treat
PP: Per-protocol
