# Supplementary material for: Effect of different weekly frequencies of Chen-style Tai Chi in elders with chronic non-specific low back pain: study protocol for a randomised controlled trial
Source: Trials. 2022 Nov 22;23:951. doi: 10.1186/s13063-022-06909-2 (PMC9682833; doi:10.1186/s13063-022-06909-2)
Supplement: Supplementary file 1 — Additional file 1. Instructions for Tai Chi. [file 13063_2022_6909_MOESM1_ESM.docx]

**Instructions for Instructors Involved in the Chen-style Tai Chi for CNLBP Programs**

The purpose of this manual is to ensure that instructors can teach Tai Chi strictly in accordance with the study protocol.

***Before Tai Chi class:***

1) One day before the class begins, you should prepare any materials related to the next day's class and write a lesson plan.

2) Rehearse the day before class as much as you'll need to do, but be prepared for the unexpected and be flexible with your study plan.

3) Twenty minutes before the class starts, you need to arrive at the teaching site and ask them how they are feeling and if they have any pain or discomfort to make sure you get a full picture of the participants' emotional, mental, and physical state.

4) Five minutes before the class begins, you need to call the roll to ensure that all participants are present, excluding those who are absent or unable to continue for special reasons.

5) During the course, you will be expected to adhere strictly to the requirements of your research plan and keep in mind the learning objectives of this course.

***During Tai Chi class:***

1) Actively interact with students to create a relaxed classroom atmosphere.

2) Show your love and professionalism for Tai Chi.

3) Introduce the cultural background of Tai Chi to increase students' interest in Tai Chi in the guided learning class.

4) Make sure participants understand the content of the course.

5) You should always get feedback from participants on how satisfied they are with the course and how they feel about the course experience.

6) Avoid conversations that are not related to the course if possible.

7) Briefly review class material from previous classes and summarize what the patients have learned in the previous weeks leading up to that class. Ask questions to ensure that participants understand the material and also offer them an opportunity to ask questions.

8) Give participants a brief introduction of today's lesson.

6) Be proficient at leading warm-ups and stretching exercises as well as cooling-down exercises.

7) Incorporate Tai Chi principles in your lesson (for example, breathing technique and Qi-building methods to improve internal energy, relaxation with visualization, meditation with movement, maintain an upright and relaxed posture at all times).

8) Use a step-by-step teaching method to teach movement, gradually increasing the difficulty of the lesson.

9) Try different teaching methods to make the class more interesting.

10) Integrate previous Tai Chi knowledge into the new class.

***After Tai Chi Class:***

1) Review what was taught during that class period, not including self-practice of TC.

2) Encourage participants to persevere during the intervention.

3) Encourage participants to form groups and supervise each other to improve their compliance.

4) Let participants look forward to the next class.

**Check List for Tai Chi Instructors**

| **Coach Number** |  | **Course Name** |  |
| --- | --- | --- | --- |
| **Course Number** |  | | |
| **Course Date** |  | | |
|  | | | **Yes No** |
| **Did you** follow your teaching protocol? | | |   |
| **Did you** review content from the previous classes at the beginning of class? | | |   |
| **Did you** let participants know what you are going to teach in the current session? | | |   |
| **Did you** incorporate the principles of Tai Chi into your teaching? | | |   |
| **Did you** review the contents of each lesson at the end of each lesson? | | |   |
| **Did you** meet your teaching session goal for today? | | |   |
| **Do you** know why the participants missed class? | | |   |
| **Did you** have difficulty communicating with some of the participants? | | |   |
| **Did you** make sure that all patients understood the content of lesson? | | |   |
| **Have you** developed a respectful relationship with your participants? | | |   |

Comments: ___________________________________________________________

Signature ________________ Date______________

| **Tai Chi Warm-ups**  10 minutes | - Warm-up exercises (stretching and review of Tai Chi movements from last lesson) |
| --- | --- |
| **Chen-Style Tai Chi**  40 minutes | - Tai Chi movements   Chen-style Tai Chi of sixteen forms |
| **Cool down**  10 minutes | - Stretching - Breathing Relaxation |

**Example of Tai Chi Class Structure**

**NOTE:**

**Warm-up:** Stretching: The core part of the warm-up will consist of exercises that encompass a variety of seated-and-standing combined stretches involving upper body (neck, upper back, shoulder, chest, and arm), lower extremities (quadriceps, hamstring/calf, and hip), and gentle and slow trunk rotations. Also included will be deep abdominal breathing exercises that emphasize inhaling and exhaling to maximum capacity, as well as progressive relaxation of major muscle groups. Besides, the warm-up exercise also includes reviewing the tai chi movements we learned in last class.

**Chen-Style Tai Chi:** Participants need to practice the following Tai Chi movements: 1) Commencing Form; 2) Buddha’s Warrior Attendant Pounds Mortar; 3) Tuck in Robes; 4) Single Whip; 5) Wave Hands Like Clouds; 6) Double Push Palms; 7) Step Back and Whirl Arms on Both Sides; 8) White Crane Spreads Wings; 9) Diagonal line spread step; 10) Deflect through The Back; 11) the Chopping Hand; 12)Strike Fist; 13) Six Seals and Four Closings; 14) Single Whip and Body Defending Punches; 15) Turn-back and Buddha’s Warrior Attendant Pounds Mortar; 16) Closing Form;

**Cool down:** Breathing Relaxation: 1) Participants sit on a cushion and inhale slowly and rhythmically, filling their chest with air. Breathing should be even, comfortable and rhythmic. 2) Participants inhale to fill their chests with air and pause to feel relaxed and comfortable, careful not to hold their breath. 3) Participants exhale slowly and fully, so that their shoulders, chest, etc., feel comfortable. Be careful not to exhale too fast.

Stretching: Same stretching routine as the warm-up part.
