# Supplementary material for: Effect of different weekly frequencies of Chen-style Tai Chi in elders with chronic non-specific low back pain: study protocol for a randomised controlled trial
Source: Trials. 2022 Nov 22;23:951. doi: 10.1186/s13063-022-06909-2 (PMC9682833; doi:10.1186/s13063-022-06909-2)
Supplement: Supplementary file 4 — Additional file 4. Sample size calculation. [file 13063_2022_6909_MOESM4_ESM.docx]

**The process of calculation in PASS 15.0 software**


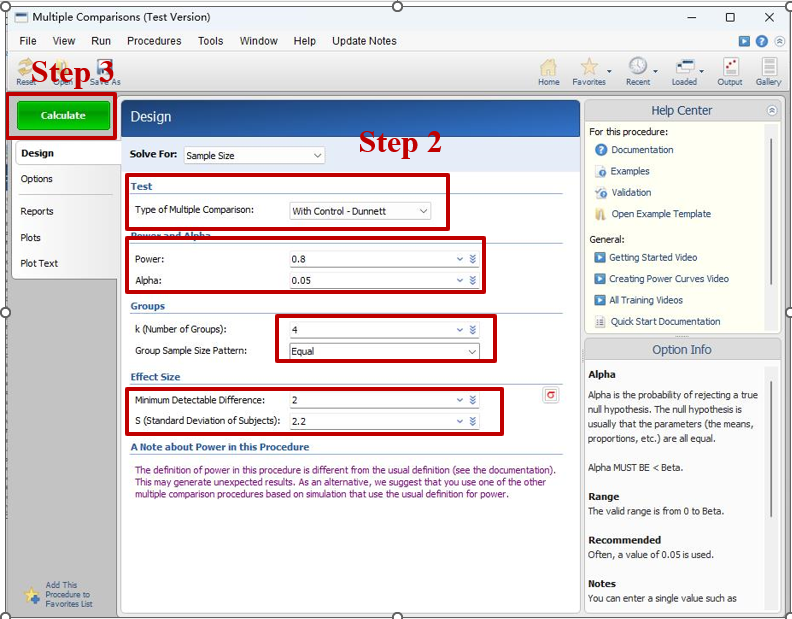

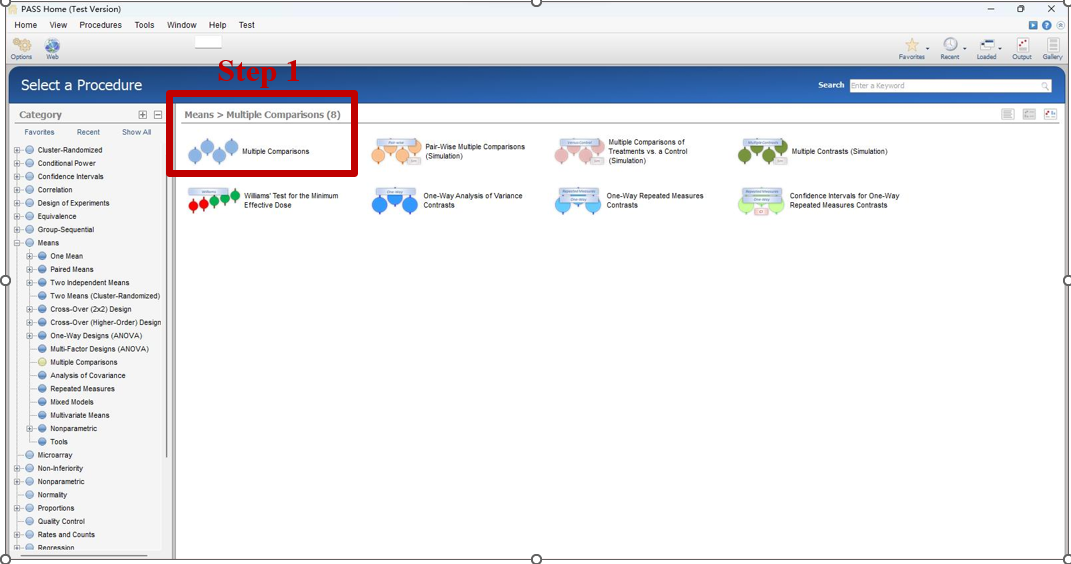


**Step 4 Obtaining the results of calculation**

PASS 2022/10/25 19:11:41 1

**Multiple Comparisons**

**Numeric Results for Multiple Comparison Test: Dunnett (With Control)**

**Average Minimum Standard**

**Size Total Detectable Deviation**

**Power (n) k N Alpha Beta Difference (S) Diff / S**

0.8330 60.00 4 240 0.0500 0.1670 2.00 2.20 0.9091

**References**

Hsu, Jason. 1996. Multiple Comparisons: Theory and Methods. Chapman & Hall. London.

**Report Definitions**

Power is the probability of rejecting a false null hypothesis. It should be close to one.

n is the average group sample size.

k is the number of groups.

Total N is the total sample size of all groups combined.

Alpha is the probability of rejecting a true null hypothesis. It should be small.

Beta is the probability of accepting a false null hypothesis. It should be small.

The Minimum Detectable Difference between any two group means.

S is the within group standard deviation.

Diff / D is the ratio of Min. Detect. Diff. to standard deviation.

**Summary Statements**

In a single factor ANOVA study, sample sizes of 60, 60, 60, and 60 are obtained from the 4

groups whose means are to be compared. The total sample of 240 subjects achieves 83% power to

detect a difference of at least 2.00 using the Dunnett (With Control) multiple comparison test

at a 0.0500 significance level. The common standard deviation within a group is assumed to be

2.20.

**Dunnett Test Details**

**Percent Minimum**

**n of Detectable Standard**

**Group n Total N Alpha Power Difference Deviation**

1 60 25.00 0.0500 0.8330 2.00 2.20

2 60 25.00

3 60 25.00

Control 60 25.00

Total 240 100.00

**Dropout-Inflated Sample Size**

**Dropout-**

**Average Inflated Expected**

**Group Enrollment Number of**

**Sample Size Sample Size Sample Size Dropouts**

**n Group Dropout Rate Ni Ni' Di**

60.00 1 - 4 20% 60 75 15

Total 240 300 60

PASS 2022/10/25 19:11:41 2

**Multiple Comparisons**

**Definitions**

n is the average group sample size.

Group lists the group numbers.

Dropout Rate (DR) is the percentage of subjects (or items) that are expected to be lost at random during the

course of the study and for whom no response data will be collected (i.e. will be treated as "missing").

Ni is the evaluable sample size for each group at which power is computed. If Ni subjects are evaluated out of

the Ni' subjects that are enrolled in the study, the design will achieve the stated power.

Ni' is the number of subjects that should be enrolled in each group in order to end up with Ni evaluable

subjects, based on the assumed dropout rate. After solving for Ni, Ni' is calculated by inflating Ni using

the formula Ni' = Ni / (1 - DR), with Ni' always rounded up. (See Julious, S.A. (2010) pages 52-53, or

Chow, S.C., Shao, J., and Wang, H. (2008) pages 39-40.)

Di is the expected number of dropouts in each group. Di = Ni' - Ni.

**Procedure Input Settings**

**Autosaved Template File**

C:\Users\wanru\Documents\PASS 15\Procedure Templates\Autosave\Multiple Comparisons - Autosaved 2022_10_25-19_11_43.t157

**Design Tab**

Solve For: Sample Size

Type of Multiple Comparison: With Control - Dunnett

Power: 0.8

Alpha: 0.05

k (Number of Groups): 4

Group Sample Size Pattern: Equal

Minimum Detectable Difference: 2

S (Standard Deviation of Subjects): 2.2

**Options Tab**

Search Precision: 0.000001
