# Supplementary material for: Effect of different weekly frequencies of Chen-style Tai Chi in elders with chronic non-specific low back pain: study protocol for a randomised controlled trial
Source: Trials. 2022 Nov 22;23:951. doi: 10.1186/s13063-022-06909-2 (PMC9682833; doi:10.1186/s13063-022-06909-2)
Supplement: Supplementary file 3 — Additional file 3. Informed consent document. [file 13063_2022_6909_MOESM3_ESM.zip › Additional File 3 Informed consent document (Original)R2.doc]

方案名称：（不同频率的陈式太极拳对老年慢性非特异性腰痛患者干预疗效的随机对照研究）

**知情同意书**

**(ICF Template)**

伦理批件编号：CSMZ2022002

研究机构：长沙民政职业技术学院

主要研究者（PI）：曹玛丽

**请您仔细阅读以下内容**

您将被邀请参加一项临床研究。本知情同意书提供给您一些信息以帮助您决定是否参加此项临床研究。请您仔细阅读，如有任何疑问可以随时向负责该项研究的研究者提出，研究人员将为您详细解答。您可以根据自己的情况作出决定，您将有充分的时间进行考虑。

**一、研究目的**

背景意义：慢性腰痛的高发病率至今仍是全球一个重要的公共健康问题，尤其是老年人。研究发现，太极拳这种身心运动被认为是缓解老年慢性腰痛患者症状的有效手段。但目前很少研究太极拳治疗老年人慢性腰痛的剂量效应关系。此外，此类多存在一些明显的局限性，如样本量小，异质性高，缺少重要的测量指标。因此本研究希望通过观察不同运动频率（每周1次、3或5次，持续12周）的陈式太极拳训练对社区老年人慢性腰痛的影响，为太极运动预防和改善慢性非特异腰痛提供科学依据。

**二、研究过程**

如果您同意参与这项研究，我们将对每位受试者进行编号，建立档案。在研究前我们会以问卷的形式采集您的部分个人信息，研究过程中我们将对您进行为期12周的运动干预。试验结束后也会进行在3个月后追踪您的情况。

**三、纳入标准和排除标准**

3.1纳入标准：

1、右利手

2、年龄不小于60岁

3、诊断为CNLBP至少3个月，符合美国国立卫生研究院(NIH)的CNLBP定义

4、视觉模拟评分（VAS）≥4

5、简易精神状态检查(MMSE)评分≥24分

6、过去6个月无规律运动及太极拳训练史

7、能独立行走，具有参与太极训练的活动能力

3.2排除标准：

1、强制性脊柱炎、恶性肿瘤、椎体骨折、脊柱感染等引起的特异性腰痛

2、限制参加太极训练的严重疾病，包括痴呆、神经系统疾病、有症状的心脏或血管疾病(心绞痛、周围血管疾病、充血性心力衰竭和严重高血压)、最近的中风、严重的胰岛素依赖性糖尿病、精神疾病、肾脏疾病、肝脏疾病、活动性癌症或贫血

3、在过去一年内曾服用过止痛药物或定期接受物理治疗

4、在过去一年内参加过类似的研究，并可能对研究结果产生影响

5、计划在试验期间永久搬离该地区

**四、研究风险与不适**

对于您来说，所有的信息将是保密的。在实验过程中会我们会运动进行干预，我们承诺此种运动方式不涉及侵入性或危害生命。如果您在试验过程中，您有任何不适，我们将中止试验。

**五、参加研究受益**

本试验会为您进行专业的行为学评估与运动干预来探究您腰痛的缓解情况，虽然我们无法保证这项运动可以给您带来直接好处，但您的参与可能会给未来遭受同样痛苦的患者带来益处。

**六、相关费用**

参加本研究不需要承担任何费用。您将免费接受所有研究检查和程序。同时实验结束后我们会根据您试验中的配合程度，将给予您相应的经济补贴。

**七、受试者职责**

作为研究受试者，您有以下职责：提供有关自身病史和当前身体状况的真实情况；告诉研究者自己在本次研究期间所出现的任何不适和服用的药物等；告诉研究者自己在最近是否曾参与其他研究，或目前正参与其他研究。
**八、自愿参加**

本研究均秉承尊重参与者及自主选择的原则。因此，您可以选择不参加本项研究，或者在任何时候通知研究者要求退出研究，您的数据将不纳入研究结果，您的任何权益不会因此而受到影响。

如果您没有遵守研究计划，未按规定进行疼痛感知而影响试验评估或者有任何其它原因，研究者可以终止您继续参与本项研究。

**九、隐私保密**

如果您决定参加本项研究，您参加试验及在试验中的个人资料均属保密。您的资料将以研究编号数字而非您的姓名加以标识。可以识别您身份的信息将不会透露给研究小组以外的成员，除非获得您的许可。所有的研究成员和研究申办方都被要求对您的身份保密。您的档案将保存在有锁的档案柜中，仅供研究人员查阅。为确保研究按照规定进行，必要时，政府管理部门或伦理审查委员会的成员按规定可以在研究单位查阅您的个人资料。这项研究结果发表时，将不会披露您个人的任何资料。
**十、受试者损伤的处理**

此研究不涉及损伤，也不会对身体造成任何损害。

**十一、其他**

本次研究相关信息以及知情同意书已通过本研究机构伦理审查委员会审查。试验过程中有任何违反研究方案的情况，您可以直接向长沙民政职业技术学院伦理委员会投诉。联系电话：18751242707，电子邮箱: 824733203@qq.com

如果自愿参加本研究，您需要签署知情同意书证明您已经了解研究的信息并自愿参加本项研究。

您可随时了解与本研究有关的信息资料和研究进展，如果您有与本研究有关的问题，或您在研究过程中发生了任何不适与损伤，或有关于本项研究参加者权益方面的问题您可以通过电话或邮箱与曹玛丽联系。

**受试者知情同意书签字页**

（1）我已经阅读了本知情同意书。

（2）我有机会提问而且所有问题均已得到解答。

（3）我理解参加本项研究是自愿的。

（4）我可以选择不参加本项研究，或者在任何时候通知研究者后退出而不会遭到歧视或报复，我的任何医疗待遇与权益不会因此而受到影响。

（5）如果我需要其它治疗，或者我没有遵守研究计划，或者发生了与研究相关的损伤或者有任何其它原因，研究医师可以终止我继续参与本项研究。

（6）我将收到一份签过字的“知情同意书”副本。

受试者（或代理人） 签名：______________

联系电话 ：______________

签名日期：	年	月	日

我确认已向患者解释了本试验的详细情况，包括其权利以及可能的获益和风险。

研究者签名：_______________

联系电话：______________

签名日期：	年	月	日
