# Supplementary material for: Effect of different weekly frequencies of Chen-style Tai Chi in elders with chronic non-specific low back pain: study protocol for a randomised controlled trial
Source: Trials. 2022 Nov 22;23:951. doi: 10.1186/s13063-022-06909-2 (PMC9682833; doi:10.1186/s13063-022-06909-2)
Supplement: Supplementary file 3 — Additional file 3. Informed consent document. [file 13063_2022_6909_MOESM3_ESM.zip › Additional File 3 Informed consent document (Translation)R2.doc]

Project number：（Effect of different weekly frequencies of Chen-style Tai Chi in elders with chronic non-specific low back pain: Study protocol for a randomised controlled trial）

**Informed Consent Form**

Ethical Approval Number: CSMZ2022002

Research institution: Changsha Social Work College

Principal Investigator (PI): Mali Cao

**Please read the following carefully**

You will be invited to participate in a clinical study. This informed consent provides you with information to help you decide whether to participate in this clinical study. Please read it carefully. If you have any questions, please feel free to ask the researcher in charge of the study, and the researcher will give you detailed answers. You can make the decision according to your own circumstances and you will have plenty of time to consider it.

**1. Study purpose**

The high incidence of chronic low back pain remains an important global public health problem, especially among the elderly. The study found that Tai Chi, a mind-body exercise, is considered to be an effective means of relieving symptoms in elderly patients with chronic low back pain. However, there are few studies on the dose-response relationship of Tai Chi in the treatment of chronic low back pain in the elderly. In addition, there are some obvious limitations, such as small sample size, high heterogeneity, and lack of important measurement indicators. Therefore, this study aims to provide a scientific basis for Tai Chi exercise to prevent and improve chronic non-specific low back pain by observing the effects of Chen style Tai chi training with different exercise frequencies (once a week, 3 or 5 times a week, for 12 weeks) on chronic low back pain in the elderly in the community.

**2. Study process**

If you agree to participate in this study, we will number each subject and establish a file. Before the study, we will collect some of your personal information in the form of a questionnaire, and during the study, we will conduct a 12-week exercise intervention for you. You will be followed up after 3 months.

**3. Inclusion criteria and exclusion criteria**

3.1 Inclusion criteria

(1) Right-handed;

(2) Adults aged 60 years or above;

(3) Diagnosed with CNLBP for at least 3 months in line with the CNLBP definition of the National Institutes of Health (NIH)

(4) Visual Analog Scale (VAS) score ≥ 4;

(5) Mini-mental Status Examination (MMSE) score ≥ 24

(6) No habitual regular exercise and TC training history in the past 6 months;

(7) Able to ambulate independently and participate in TC training.

3.2 Exclusion criteria：

(1) LBP is caused by mandatory spondylitis, malignant tumors, vertebral fractures, spinal infection, etc

(2) Serious diseases that limit ability to participate in TC training, including dementia, neurological disease, symptomatic heart or vascular disease (angina, peripheral vascular disease, congestive heart failure, and severe hypertension), recent stroke, severe insulin-dependent diabetes mellitus, psychiatric disease, renal disease, liver disease, active cancer or anaemia

(3) Having analgesic drugs or regular physical therapy programs for CNLBP within the past year

(4) Participating in a similar within the past year and could have an impact on the results of this study

(5) Planning to move permanently from the area during the trial period.

**4. Study risk and discomfort**

All information will be confidential to you. During the study, we will exercise intervention, and we promise that such exercise will not involve invasive or life-threatening. If you have any discomfort during the test, we will stop the study.

**5. Study benefits**

This trial will provide you with a professional behavioral assessment and exercise intervention to explore the relief of your low back pain. Although we cannot guarantee that this exercise will bring you direct benefits, your participation may bring benefits to future patients suffering from similar pain.

**6. Study expense**

There is no cost to participate in this study. You will receive all study examinations and procedures free of charge. At the same time, after the experiment, we will give you the corresponding economic subsidy according to the degree of cooperation in the study.

**7. Subject's responsibilities**

As a study subject, you have the following responsibilities: to provide truthful information about your medical history and current medical condition; Inform the researcher of any discomfort and medication they have taken during the study; Tell the researcher whether you have recently participated in other studies or are currently participating in other studies.
**8.** **Voluntary participation**

The principle of respect for participants and the independent choice was adhered to in this study. Therefore, you may choose not to participate in the study, or at any time notify the investigator to withdraw from the study, and your rights and interests will not be affected.

The investigator may terminate your participation in the study if you fail to comply with the study plan, interfere with the evaluation of the study by failing to perform the prescribed pain perception, or for any other reason.

**9. Data privacy**

If you decide to participate in this study, your personal data will be kept confidential. Your information will be identified by a study number rather than your name. Information that identifies you will not be disclosed to other members of the research team unless you give your permission. All study members and sponsors are required to keep your identity confidential. Your files will be kept in a locked filing cabinet for researchers to access only. In order to ensure that the research is conducted in accordance with the regulations, members of the government management department or the ethics review committee may access your personal data in the research unit if necessary. No personal information about you will be disclosed when the results of this study are published.

**10. Management of subject injury**

There was no injury involved in the study, nor was there any physical harm.

**11. Other**

Information related to this study and informed consent have been reviewed by the ethics Review Committee of this research institution. If there is any violation of the research protocol during the test, you can directly complain to the ethics Committee of Changsha Social Work College. Contact number: +8618751242707, email: 824733203@qq.com.

If you are willing to participate in this study, you will need to sign an informed consent to prove that you have understood the study information and volunteered to participate in this study.

You can keep track of the information and progress of this study at any time. If you have any questions related to this study, have any discomfort or injury during the study, or have any questions about the rights and interests of participants in this study, you can contact Mali Cao by phone or email.

**Informed Consent · Consent Signature Page**

（1）I have read this informed consent form.

（2）I had the opportunity to ask questions and all of them were answered

（3）I understand that participation in this study is voluntary

（4）I can choose not to participate in this study, or withdraw at any time after notifying the investigator without being subjected to discrimination or retaliation, and my medical treatment and rights will not be affected.

（5）If I need other treatments, don't follow the study play, or for any other reason, the study physician can terminate my participation in the study.

（6）I will receive a signed copy of the informed consent form

I have understood the above situation and have decided to participate in this

clinical study with my written consent.

Participant (or agent) signature：______________

Telephone number：______________

Signature date：______________

I confirm that the details of this study, including their rights, benefits, and

possible risks, have been explained to participants.

Investigator signature：______________

Telephone number：______________

Signature date：______________
